# Hereditary leukoencephalopathy with axonal spheroids: a spectrum of phenotypes from CNS vasculitis to parkinsonism in an adult onset leukodystrophy series

**DOI:** 10.1136/jnnp-2015-310788

**Published:** 2015-05-02

**Authors:** David S Lynch, Zane Jaunmuktane, Una-Marie Sheerin, Rahul Phadke, Sebastian Brandner, Ionnis Milonas, Andrew Dean, Nin Bajaj, Nuala McNicholas, Daniel Costello, Simon Cronin, Chris McGuigan, Martin Rossor, Nick Fox, Elaine Murphy, Jeremy Chataway, Henry Houlden

**Affiliations:** 1 Department of Molecular Neuroscience, The National Hospital for Neurology and Neurosurgery, UCL Institute of Neurology, Queen Square, London, UK; 2 The Leonard Wolfson Experimental Neurology Centre, The National Hospital for Neurology and Neurosurgery, UCL Institute of Neurology, London UK; 3 Division of Neuropathology and Department of Neurodegenerative Disease, The National Hospital for Neurology and Neurosurgery, UCL Institute of Neurology, Queen Square, London, UK; 4 Aristotle University of Thessaloniki, Thessaloniki, Greece; 5 Department of Neuropathology, Addenbrooke's Hospital, Cambridge, UK; 6 Department of Neurology, Queens Medical Centre, Nottingham, UK; 7 Department of Neurology, Cork University Hospital, Wilton, Cork, Ireland; 8 University College Dublin, St. Vincent's University Hospital, Dublin, Ireland; 9 Department of Neurodegeneration, Dementia Research Centre, London, UK; 10 Department of Neuroinflammation, The National Hospital for Neurology and Neurosurgery, UCL Institute of Neurology, Queen Square, London, UK; 11 Neurogenetics Laboratory, The National Hospital for Neurology and Neurosurgery, UCL Institute of Neurology, Queen Square, London, UK

## Abstract

**Background:**

Hereditary diffuse leukoencephalopathy with neuroaxonal spheroids (HDLS) is a hereditary, adult onset leukodystrophy which is characterised by the presence of axonal loss, axonal spheroids and variably present pigmented macrophages on pathological examination. It most frequently presents in adulthood with dementia and personality change. HDLS has recently been found to be caused by mutations in the colony stimulating factor-1 receptor (*CSF1R*) gene.

**Methods:**

In this study, we sequenced the *CSF1R* gene in a cohort of 48 patients from the UK, Greece and Ireland with adult onset leukodystrophy of unknown cause.

**Results:**

Five pathogenic mutations were found, including three novel mutations. The presentations ranged from suspected central nervous system (CNS) vasculitis to extrapyramidal to cognitive phenotypes. The case histories and imaging are presented here, in addition to neuropathological findings from two cases with novel mutations.

**Conclusion:**

We estimate that *CSF1R* mutations account for 10% of idiopathic adult onset leukodystrophies and that genetic testing for *CSF1R* mutations is essential in adult patients presenting with undefined CNS vasculitis or a leukodystrophy with prominent neuropsychiatric signs or dementia.

## Introduction

Hereditary diffuse leukoencephalopathy with neuroaxonal spheroids (HDLS) is an autosomal dominant, adult onset leukodystrophy which typically presents with early onset cognitive or personality change. It is characterised by a distinct neuropathological appearance consisting of axonal loss in the cerebral white matter, axonal spheroids and variably present pigmented microglia. In 2011, it was discovered that heterozygous mutations in the colony stimulating factor-1 receptor (*CSF1R*) gene cause HDLS.^1^ In addition, it was shown that pigmented orthochromatic leukodystrophy (POLD) is also caused by *CSF1R* mutations and that POLD and HDLS exist on a spectrum.^2^ Previous studies have estimated that *CSF1R* mutations account between 10% and 25% of adult onset leukodystrophies, depending on the population studied.^3^ ^4^

The clinical phenotype of patients with HDLS is variable, but the most common symptoms include cognitive decline, personality change and depression. Additional symptoms occur frequently and include parkinsonism, spasticity and seizures. Median age of onset is 45 years, although patients with onset as young as 18 have been described. Median life expectancy is 6 years but this is also variable, and some patients have survived for up to 29 years after symptom onset.^3^ ^5^

**Figure 1:**
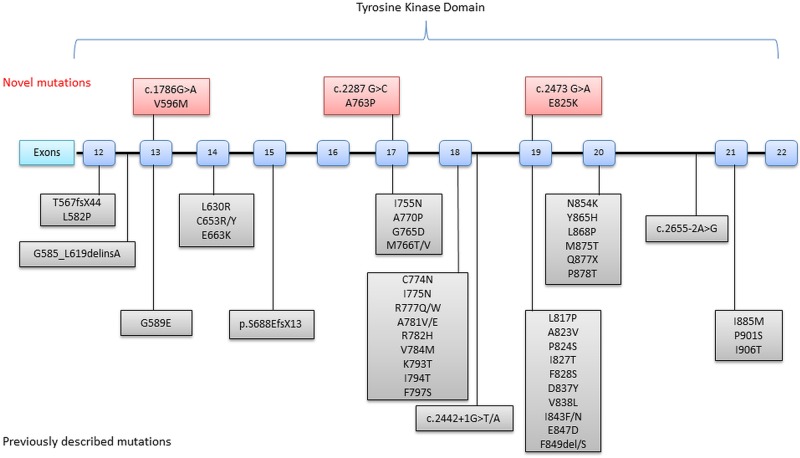
Schematic representation of the colony stimulating factor-1 receptor (CSF1R) gene illustrating new and previously described mutations.

All mutations identified to date have been found in the tyrosine kinase domain of the protein (exons 12–21) with exons 18, 19 and 20 containing the majority of the mutations (see figure 1 and online supplementary table S1). CSF1R is a cell surface receptor that is highly expressed on cells of the myeloid lineage including the microglia of the central nervous system (CNS).^6^ It is activated by the cytokines colony stimulating factor-1 (CSF1) and interleukin-34. The receptor consists of an extracellular ligand binding domain, a transmembrane domain and an intracellular tyrosine kinase domain.^6^ Binding of CSF1 to the CSF1R receptor results in receptor homodimerisation and the autophosphorylation of a number of tyrosine residues in the intracellular domain. This is followed by activation of several signalling pathways including Src,^7^ AKT, Erk and phospholipase C-γ.^8^ CSF1R activation therefore regulates microglial survival, proliferation and differentiation.

## Methods

### Patients

Patients with adult onset (>16 years) leukodystrophy of unknown cause were recruited non-consecutively from the National Hospital for Neurology and Neurosurgery, London; University College Cork, Ireland; St Vincent's University Hospital, Ireland; and Aristotle University of Thessaloniki, Greece, as part of the inception cohort of the adult onset leukodystrophy group multidisciplinary clinical service.^9^ ^10^ The main inclusion criterion was MRI white matter abnormalities consistent with leukodystrophy, that is, symmetric confluent T2 hyperintensity but excluding those with asymmetric/atypical features more likely associated with small vessel disease or multiple sclerosis. All patients had MRI and routine biochemical (including very long chain fatty acids), haematological, infectious and immune screening. Informed consent was obtained for genetic research sequencing and the project was carried out with institutional ethical approval from all centres.

### Genetic analysis

The *CSF1R* gene was sequenced in all 48 patients as follows. The entire coding region of *CSF1R* was PCR-amplified using flanking intronic primers (primer sequences available on request). The PCR product was purified and then sequenced in both directions using Big Dye Terminator V.3.1 Cycle Sequencing Kit (Applied Biosystems). Sequencing products were purified and read on an ABI 3730 DNA Analyzer (Applied Biosystems). Sequences were analysed using Seqscape V.3 software (Applied Biosystems). Variants are described with reference to Ensembl Transcript ENST00000286301 of the *CSF1R* gene. In silico prediction was performed using Polyphen-2^11^ and Provean.^12^

### Neuropathological analysis

Formalin-fixed, paraffin-embedded tissue was cut to 4 µm thick sections (14 µm for Luxol fast blue), mounted on glass slides and stained with routine H&E, periodic acid-Schiff (PAS) and Luxol fast blue/cresyl violet histochemical stains. Sections were examined by immunohistochemistry with the following antibodies: glial fibrillar acid protein (GFAP) (polyclonal, 1:2500, Dako), phosphorylated neurofilaments (clone SMI31, 1:5000, Sternberg), neurofilament cocktail (clone 2F11, 1:500, Dako/Cappel), myelin basic protein (clone SMI94, 1:2000, Sternberger), amyloid precursor protein (clone 22C11, 1:800, Chemicon/Millipore), amyloid-β (clone 6F3D, 1:100, Dako), ubiquitin (polyclonal, 1:1200, Dako), p62 (3/P62LCK Ligand, 1:100, BD Transduction), α-synuclein (clone KM51, 1:50, Leica/Novocastra), hyperphosphorylated τ (clone AT8, 1:1200, INNOGENETICS), TDP-43 (clone 2E2-D3, 1:3000, Abnova), CD68 (clone PG-M1, 1:100, Dako), CD3 (LN10, 1:100, Leica/Novocastra), CD20 (clone 7D1, 1:200, Dako). Immunohistochemistry was carried out on a BondMax autostainer (Leica Microsystems) using 3,3-diaminobenzidine as chromogen. Appropriate positive controls were used for all immunohistochemical studies. Negative controls were treated identically except that the primary antibody was omitted. Negative control sections were examined for pigment deposits under bright light, including assessment of digital negative image with total inversion of the light (LEICA SCN400 scanner at ×40magnification and 65% image compression setting (LEICA UK)).

## Results

We sequenced the *CSF1R* gene in the 48 patients presenting with adult onset leukodystrophy of unknown cause from the UK, Greece and Ireland and identified five patients carrying mutations in the gene (including 3 novel mutations), indicating that *CSF1R* mutations account for approximately 10% of adult onset leukodystrophies in our cohort. A summary of identified mutations and clinical spectrum is given in table 1. Sequence alignment and in silico pathogenicity predictions for the novel V596M, A763P and E825K mutations are provided in online supplementary figure S1. We did not identify any discriminating clinical feature that could predict whether a CSF1R mutation would be found (see online supplementary table S2 for a summary of the negative cases).

**Table 1:** Summary of the clinical characteristics and investigations of patients with CSF1R mutations

Case	Mutation	Family history	Age at onset	Age at death	Initial symptoms	Additional symptoms	MRI	Neuropathology
1	c.1786G>A p. V596M	No	25	35	Sensory symptoms, cognitive decline	Supranuclear gaze palsy, seizures, pyramidal signs	WML, changing DWI positivity suggesting vasculitis	Subcortical AS and astrogliosis, pigmented macrophages
2	c.2287G>A p. A763P	No	45	51	Depression, personality change	Parkinsonism, apraxia, seizures	WML	Myelin/axonal loss in deep WM with frequent AS and pigmented macrophages
3	c.2473G>A p. E825K	Yes	42	Alive	Ataxia, cognitive decline	Apraxia, tremor	WML, atrophy, TCC	NA
4	c.2442+1 G>A	No	43	Alive	Depression	Parkinsonism, apraxia	WML, atrophy, TCC	NA
5	c.1987G>A E633K	No	29	NA	Falls, cognitive decline	Parkinsonism, apraxia, pyramidal signs	WML, cortical atrophy	NA

AS, axonal spheroids; CSF1R, colony stimulating factor-1 receptor; DWI, diffusion weighted imaging; NA, not applicable; TCC, thinning of the corpus callosum; WML, white matter lesions.

### Case 1

This patient had struggled at school with learning difficulties and developed sensory symptoms in the limbs at age 25 years. She was involved in a car accident at age 31 years, following which she developed mild cognitive symptoms and executive dysfunction. By age 35 years, there was progressive immobility, cognitive decline and urinary incontinence. Aged 36 years she rapidly deteriorated over a 3-month period, with anarthria, loss of mobility and worsening dementia. Her father had died of motor neuron disease aged 65 years after an 18-month illness. Her mother had been treated for alcoholism and was still alive with a number of physical and cognitive problems.

Examination revealed a supranuclear gaze palsy and a brisk jaw jerk. There was an asymmetric spastic quadriparesis and left-sided inattention. She was abulic with a frontal subcortical pattern of cognitive impairment. Mini-Mental State Examination was 8/30.

**Figure 2:**
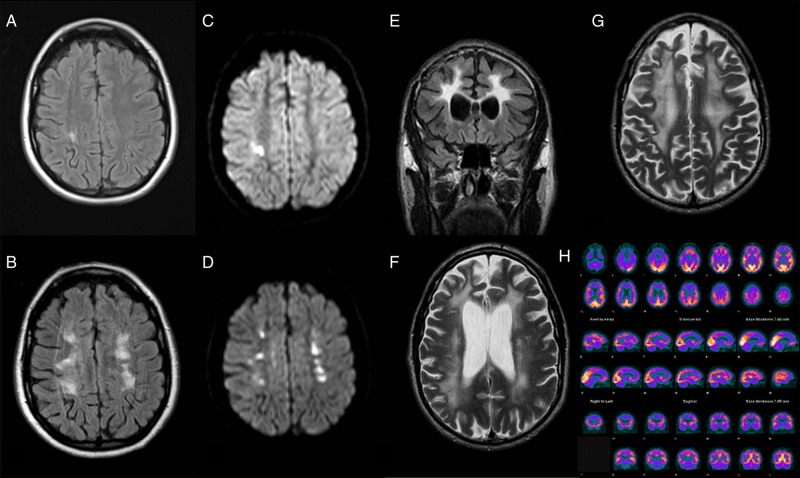
FLAIR (A) and diffusion weighted imaging (DWI) (C) imaging of case 1 aged 34 years demonstrating restricted diffusion and FLAIR hyperintensity in the right parietal lobe. At age 36 imaging findings had progressed as can be seen in FLAIR (B) and DWI (D) images. The number of lesions showing restricted diffusion raised the possibility of central nervous system vasculitis. Case 2 imaged at age 48 shows extensive white matter hyperintensities on FLAIR (E) and T2-weighted imaging (F and G). There is reduced fluorodeoxyglucose (FDG) uptake in the frontal and right parietal lobes as seen in this FDG-positron emission tomography scan (H).

Neuroimaging: The first MRI of the brain at age 31 years was reported as showing an acute parietal infarct with restricted diffusion; however, subsequent imaging showed progressive extensive signal change with patches of restricted diffusion and volume loss in the superficial and deep white matter with relative sparing of the subcortical U fibres (see figure 2). There was disproportionate volume loss and T2-weighted hyperintensity of the splenium of the corpus callosum extending to the posterior limb of both internal capsules and corticospinal tracts at the level of the mid brain. There was no abnormal contrast enhancement and MR angiogram was normal. These imaging findings were thought to be suspicious for cerebral vasculitis.

CSF examination, nerve conductions studies and echocardiogram were normal. EEG showed generalised background slowing. A right frontal brain biopsy was performed to investigate the possibility of cerebral vasculitis.

**Figure 3:**
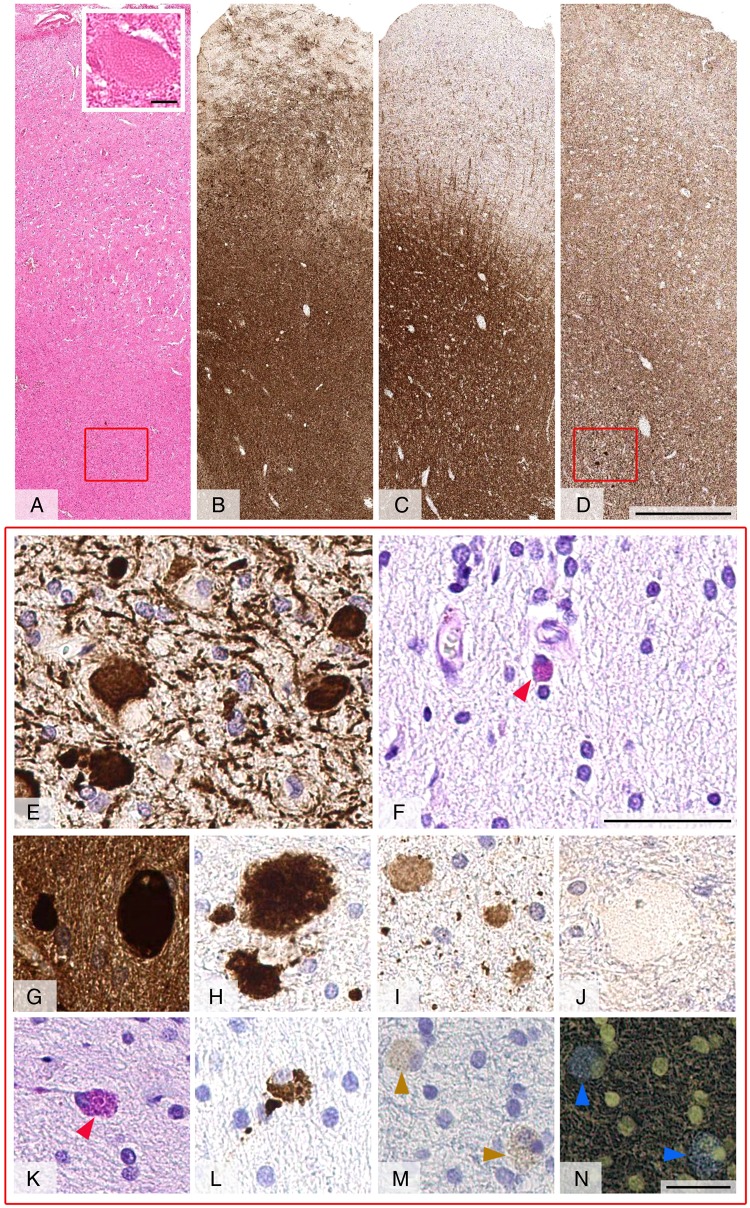
Full thickness brain biopsy of case 1. The H&E stained section (A) shows frequent axonal swellings in the subcortical white matter (inset). Immunostaining for glial fibrillar acid protein (B) reveals a severe chronic fibrillar and reactive stellate astrogliosis in the subcortical white matter and in the cortex. Immunostaining for myelin basic protein with SMI94 antibody (C) and of axons with SMI31 antibody (D) reveals no apparent myelin or axon loss. SMI31 immunoreactive axonal spheroids are frequent (E) while periodic acid-Schiff (PAS) positive pigmented glial cells (F, red arrowhead) are sparse. Axonal spheroids are positive for p62 (G), amyloid precursor protein (H) and amyloid-β (I), and negative for α-synuclein (J). Occasional scattered PAS-positive cells in the white matter (K, red arrowhead) show positive labelling for the macrophage lysosome marker CD68 (L). A negative control section (M) highlights the yellow-brown pigment in the cytoplasm of these monocyte-derived cells (brown arrowheads), which appears blue in a negative—complete colour inversion image (N, blue arrowheads). Scale bar: 1 mm in A–D, 10 µm inset in A, 50 µm in E–F, 50 µm in G–N.

Neuropathology (figure 3): Demonstrated well-preserved hexalaminar architecture of the neocortex with no obvious balloon cells (figure 3A). The leptomeninges were unremarkable. In the subcortical white matter, there were frequent eosinophilic axonal swellings, which showed positive labelling for phosphorylated neurofilaments, p62, amyloid precursor protein, amyloid-β (figure 3E, G–I) and ubiquitin. Sparse numbers of PAS and CD68 positive pigmented cells were evident in the white matter (figure 3F, K, L). Immunostaining for GFAP revealed severe reactive stellate and chronic fibrillar astrogliosis in the subcortical white matter and to a lesser extent in the cortex (figure 3B). In spite of frequent axonal spheroids, myelin pallor or significant reduction in density of axons were not apparent (figure 3C, D). There were no α-synuclein (figure 3J), TDP43 or hyperphosphorylated τ positive inclusions in the cortex or white matter, and T lymphocytes and B lymphocytes were very sparse.

The patient died suddenly of a massive pulmonary embolus aged 36.

Genetic analysis: *CSF1R* sequencing revealed a novel heterozygous c.1786G>A mutation leading to a V596M substitution in exon 13.

### Case 2

This patient presented at age 47 years with a 2-year history of depression and personality change, including increasing use of illicit drugs. He made reckless financial decisions and became violent at home. Progressive cognitive deterioration followed and within 2 years of presentation he required long-term care. He developed frequent generalised seizures following a right frontal brain biopsy and gradually became mute and uncommunicative. There was no family history of similar illness.

On examination, there was upper limb apraxia and a parkinsonian gait with prominent freezing. Later examination findings included severe rigidity in the left upper limb, a rest tremor and stimulus-sensitive myoclonus. Eye movements were affected by slow saccades, visual impersistance and a mild vertical supranuclear gaze palsy.

MRI demonstrated extensive symmetrical T2 high signal in the cerebral white matter involving the frontal, parietal and posterior temporal lobes (figure 2). The lateral and third ventricles were enlarged due to cerebral volume loss. A DaTscan was normal and fluorodeoxyglucose/positron emission tomography scan of the brain demonstrated reduced tracer uptake in the frontal and right parietal lobes. There was no contrast enhancement or diffusion weighted imaging (DWI) positivity and imaging appearances remained stable after 1 year.

**Figure 4:**
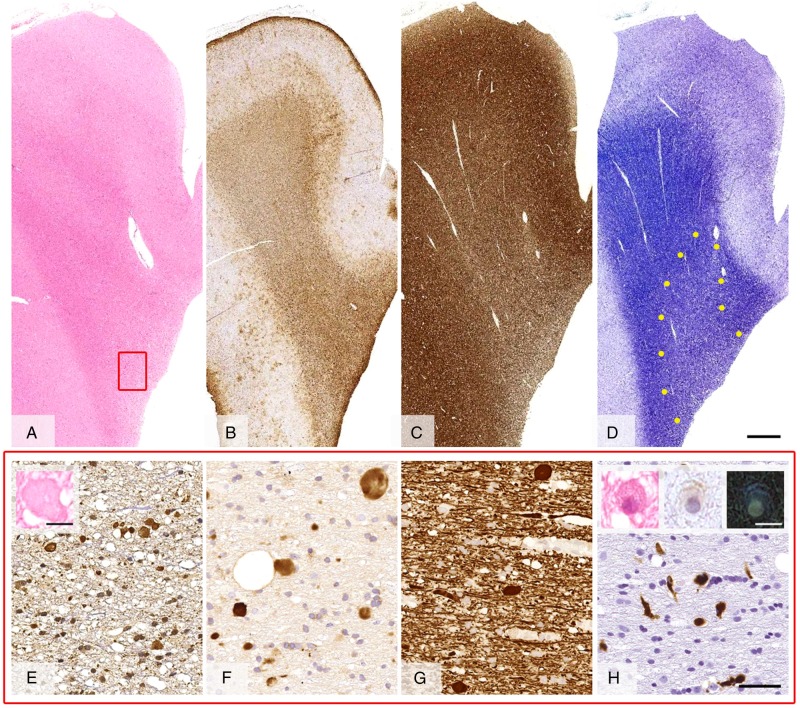
Full thickness brain biopsy of case 2. The H&E stain (A), immunostaining for glial fibrillar acid protein (B), axons (neurofilament cocktail) (C) and myelin (Luxol fast blue/cresyl violet) (D) shows a mild pallor of the myelin and reduction of axon density towards the deep white matter (separated by a yellow dotted line in D) where frequent axonal spheroids are seen (inset in E). The axonal spheroids label with antibodies for neurofilaments (E), amyloid precursor protein (F) and ubiquitin (G). Increased numbers of CD68 positive microglial cells are present in the deeper white matter (H), which show yellow-light brown cytoplasm on H&E and negative control sections and appear blue when viewed as a negative colour inversion image (insets in H). Scale bar: 1 mm in A–D, 5 µm in E–H, 10 µm insets in E and H.

Neuropathology (figure 4): A right frontal brain biopsy revealed unremarkable leptomeninges and well-preserved hexalaminar cytoarchitecture of the cortex (figure 4A). Myelin pallor and axonal loss (figure 4C, D) were evident in the deeper part of the white matter where frequent axonal spheroids and moderate numbers of pigmented cells were seen. Axonal spheroids contained neurofilaments, amyloid precursor protein and ubiquitin (figure 4E–G). Pigmented cells, which showed yellow-brown colour of the cytoplasm on H&E stained sections, showed positive labelling for CD68 (figure 4H). Immunostainings for hyperphosphorylated τ and α-synuclein were negative and amyloid-β immunoreactivity was restricted to some of the axonal spheroids.

Genetic analysis: *CSF1R* sequencing demonstrated a novel heterozygous c.2287G>A mutation resulting in an A763P substitution in exon 19.

### Case 3

The patient developed symptoms at age 42 years. The first symptom was impaired balance and unsteadiness when walking and she was referred to a neurologist for investigation of ataxia. Cognitive symptoms developed over the following 9 months with disinhibition, abulia and short-term memory impairment. Urinary incontinence also developed. There was a family history of a similar illness. The patient's mother died at age 42 years with swallowing, memory and walking difficulties. Two maternal uncles were similarly affected.

On examination, there was evidence of a global intellectual decline, with frontal and subcortical predominance. There was mild ideomotor and constructional apraxia. There was a postural and action tremor in the limbs with bilateral Gegenhalten.

Neuroimaging: MRI revealed gross white matter signal abnormality and cortical volume loss, more significant in the frontal lobe with thinning of the corpus callosum. The lateral ventricles were grossly dilated secondary to cerebral white matter loss. Follow-up MRI 2 years later showed progression of the frontal lobe white matter lesions. There was no contrast enhancement or DWI-positive lesions.

Genetic analysis: *CSF1R* sequencing revealed a novel heterozygous c.2473G>A mutation causing an E825K substitution in exon 19.

### Case 4

This 45-year-old patient worked as a manager at a hotel and presented with a 1–2-year history of depression and increasing difficulty at work. They had become withdrawn and lost confidence in their ability to solve problems, resulting in being made redundant. The family doctor treated the patient for depression without success.

On examination, there was upper limb apraxia and a parkinsonian gait. Cognitive examination revealed significant frontal lobe dysfunction and impaired short-term memory. On examination of eye movements, pursuit was normal but saccades were slow. There was no rigidity in the limbs, myoclonus or dystonia.

Neuroimaging: MRI revealed confluent, symmetrical T2 high signal in the frontal and parietal lobes. There was associated atrophy with ventricular dilation and thinning of the corpus callosum. There was relative sparing of the peritrigonal regions. There was no contrast enhancement or DWI-positive lesions. Follow-up imaging 1 year later was unchanged.

Genetic analysis: *CSF1R* sequencing revealed a heterozygous c.2442+1 G>A mutation at a splice site involving exon 18. This mutation has previously been reported to cause HDLS.^13^ In addition, functional work has shown that mutations at this splice site lead to three aberrant splice variants which exclude exon 18.^14^

### Case 5

This patient developed symptoms at the age of 29 years. Initial symptoms included falls, short-term memory loss and brief, epileptiform episodes with staring and non-responsiveness. They suffered steady cognitive decline during the 30s and by age 40 years was significantly dependent and incontinent with frequent generalised seizures. There was no family history of a similar illness.

On examination, the right upper limb was held in a flexed dystonic posture with rigidity. Bilateral grasp reflexes and rooting, suck and pout reflexes were present. Lower limb tone was symmetrically increased with sustained clonus and bilateral extensor plantar responses. There was bilateral tremor, bradykinaesia and a festinating gait.

Neuroimaging: MRI showed predominantly anterior periventricular white matter hyperintensities and global cortical atrophy. There was no contrast enhancement or DWI-positive lesions. Follow-up imaging 1 year later was unchanged.

Genetic analysis: *CSF1R* sequencing revealed a heterozygous c.1987G>A mutation causing an E633K substitution in exon 14. This mutation has been reported to cause HDLS previously in a number of reports.^13^

## Discussion

*CSF1R* mutations are part of a small but growing list of microglia-associated neurodegenerative diseases. While early reports suggested that the mutations had a dominant-negative effect, it has now been shown that *CSF1R* mutations are loss of function. Mutant CSF1R is expressed on the cell surface and can bind CSF1, form dimers and be internalised.^15^ Therefore, heterozygous mutations result in a 75% reduction in active CSF1/CSF1R dimers that can signal normally.

Appropriate CSF1R signalling may be essential not only for CNS development, but also for the health of the fully developed brain. Recently it was shown that microglia in the adult brain are actively dependent on CSF1R signalling. Inhibition of signalling through CSF1R was found to lead to the rapid depletion of almost all brain microglia via apoptosis, with repopulation occurring when inhibition was withdrawn.^16^

The importance of microglia in maintaining a healthy CNS is increasingly recognised. Interestingly, homozygous mutations in *TREM2*, another microglial cell surface receptor, cause an early onset dementia, Nasu-Hakola disease.^17^ Heterozygous variants in *TREM2* are also a significant risk factor for Alzheimer's disease (AD),^18^ and genome-wide association studies have linked variants in the microglial receptors CD33 and IRF8 with AD and multiple sclerosis, respectively.^19^ ^20^ These findings clearly point to the importance of microglia in the health of the CNS and the need to further study how microglial dysfunction leads to neuronal death.

In this study, we found that *CSF1R* mutations account for approximately 10% of adult onset leukodystrophies in a mixed cohort from the UK, Greece and Ireland. We identified a range of phenotypes, with case 1 presenting with features suggesting a CNS vasculitis; cases 2, 4 and 5 had early and late parkinsonian features; and case 3 had a more classical cognitive phenotype.

There are at least 30 different leukodystrophies that can present in adulthood, many of which have similar or even indistinguishable presentations making accurate diagnosis challenging.^9^ Presence of axonal spheroids, pigmented cells and white matter degeneration in diagnostic brain biopsies is highly variable, subject to variable regional predilection for pathology, sampling bias and disease stage. Diagnostic brain biopsies should ideally include full thickness of the cortex and underlying white matter to reduce the sampling bias. In many cases, extensive and expensive testing is required to make a diagnosis. Our study confirms previous findings that *CSF1R* mutations are a relatively common cause of adult onset leukodystrophy and that *CSF1R* mutations can lead to diverse phenotypes and can mimic many other neurological diseases, including CNS vasculitis.

As the phenotype of *CSF1R* mutations continues to be refined, we recommend that patients who present with a possible CNS vasculitis or undiagnosed adult onset leukodystrophy be screened early for mutations in *CSF1R*, and this should not be limited to patients with typical neuropsychiatric or parkinsonian presentations. The early detection of a known pathogenic *CSF1R* mutation may negate the need for a brain biopsy and its associated risks. Early genetic diagnosis in affected individuals will help guide clinician discussions around prognostication, genetic risk in other family members and reproductive counselling in families.

In addition, where a brain biopsy has been performed for a suspected CNS vasculitis and a diagnosis not achieved, the biopsy sample should be carefully examined for the presence of axonal spheroids or pigmented glia and consideration given to *CSF1R* gene sequencing.

### Sharing of data and material in this report

Genetic data, DNA samples, control data and neuropathological slides are open access for sharing with other research groups.

## Supplementary Material

Web supplement

Web table 1

Web table 2

## References

[1] Rademakers R, Baker M, Nicholson AM, et al Mutations in the colony stimulating factor 1 receptor (CSF1R) gene cause hereditary diffuse leukoencephalopathy with spheroids. Nat Genet 2011;44:200–5. 10.1038/ng.1027 22197934 PMC3267847

[2] Nicholson AM, Baker MC, Finch NA, et al CSF1R mutations link POLD and HDLS as a single disease entity. Neurology 2013;80:1033–40. 10.1212/WNL.0b013e31828726a7 23408870 PMC3653204

[3] Guerreiro R, Kara E, Le Ber I, et al Genetic analysis of inherited leukodystrophies: genotype-phenotype correlations in the CSF1R gene. JAMA Neurol 2013;70:875–82. 10.1001/jamaneurol.2013.698 23649896 PMC4204151

[4] Karle KN, Biskup S, Schüle R, et al De novo mutations in hereditary diffuse leukoencephalopathy with axonal spheroids (HDLS). Neurology 2013;81:2039–44. 10.1212/01.wnl.0000436945.01023.ac 24198292

[5] Sundal C, Lash J, Aasly J, et al Hereditary diffuse leukoencephalopathy with axonal spheroids (HDLS): a misdiagnosed disease entity. J Neurol Sci 2012;314:130–7. 10.1016/j.jns.2011.10.006 22050953 PMC3275663

[6] Stanley ER, Chitu V CSF-1 receptor signaling in myeloid cells. Cold Spring Harb Perspect Biol 2014;6:pii: a021857 10.1101/cshperspect.a021857 PMC4031967 24890514

[7] Rohde CM, Schrum J, Lee AW A juxtamembrane tyrosine in the colony stimulating factor-1 receptor regulates ligand-induced src association, receptor kinase function, and down-regulation. J Biol Chem 2004;279:43448–61. 10.1074/jbc.M314170200 15297464

[8] Yeung YG, Stanley ER Proteomic approaches to the analysis of early events in colony-stimulating factor-1 signal transduction. Mol Cell Proteomics 2003;2:1143–55. 10.1074/mcp.R300009-MCP200 12966146

[9] Ahmed RM, Murphy E, Davagnanam I, et al A practical approach to diagnosing adult onset leukodystrophies. J Neurol Neurosurg Psychiatry 2014;85:770–81. 10.1136/jnnp-2013-305888 24357685

[10] Merwick A, Ahmed R, Murphy E, et al Adult leukodystrophy: multi-disciplinary structured approach. J Neurol Neurosurg Psychiatry 2014;85:e4 10.1136/jnnp-2014-309236.81

[11] Adzhubei IA, Schmidt S, Peshkin L, et al A method and server for predicting damaging missense mutations. Nat Methods 2010;7:248–9. 10.1038/nmeth0410-248 20354512 PMC2855889

[12] Choi Y, Sims GE, Murphy S, et al Predicting the functional effect of amino acid substitutions and indels. PLoS ONE 2012;7:e46688 10.1371/journal.pone.0046688 23056405 PMC3466303

[13] Saitoh BY, Yoshida K, Hayashi S, et al Sporadic hereditary diffuse leukoencephalopathy with axonal spheroids showing numerous lesions with restricted diffusivity caused by a novel splice site mutation in the CSF1R gene. Clin Exp Neuroimmunol 2013;4:76–81. 10.1111/cen3.12076

[14] Konno T, Tada M, Tada M, et al Haploinsufficiency of CSF-1R and clinicopathologic characterization in patients with HDLS. Neurology 2014;82:139–48. 10.1212/WNL.0000000000000046 24336230 PMC3937843

[15] Pridans C, Sauter KA, Baer K, et al CSF1R mutations in hereditary diffuse leukoencephalopathy with spheroids are loss of function. Sci Rep 2013;3:3013 10.1038/srep03013 24145216 PMC3804858

[16] Elmore MR, Najafi AR, Koike MA, et al Colony-stimulating factor 1 receptor signaling is necessary for microglia viability, unmasking a microglia progenitor cell in the adult brain. Neuron 2014;82:380–97. 10.1016/j.neuron.2014.02.040 24742461 PMC4161285

[17] Paloneva J, Manninen T, Christman G, et al Mutations in two genes encoding different subunits of a receptor signaling complex result in an identical disease phenotype. Am J Hum Genet 2002;71:656–62. 10.1086/342259 12080485 PMC379202

[18] Jin SC, Benitez BA, Karch CM, et al Coding variants in TREM2 increase risk for Alzheimer's disease. Hum Mol Genet 2014;23:5838–46. 10.1093/hmg/ddu277 24899047 PMC4189899

[19] Jiang T, Yu JT, Hu N, et al CD33 in Alzheimer's disease. Mol Neurobiol 2014;49:529–35. 10.1007/s12035-013-8536-1 23982747

[20] Yoshida Y, Yoshimi R, Yoshii H, et al The transcription factor IRF8 activates integrin-mediated TGF-β signaling and promotes neuroinflammation. Immunity 2014;40:187–98. 10.1016/j.immuni.2013.11.022 24485804 PMC4105266

