# Supplementary material for: Hereditary leukoencephalopathy with axonal spheroids: a spectrum of phenotypes from CNS vasculitis to parkinsonism in an adult onset leukodystrophy series
Source: J Neurol Neurosurg Psychiatry. 2015 May 2;87(5):512–9. doi: 10.1136/jnnp-2015-310788 (PMC4853550; doi:10.1136/jnnp-2015-310788)

Supplementary Figure 1. (A) Sequence chromatograms illustrating V596M, A763P and E825K mutations. (B) Multiple species alignment showing conservation of residues for 3 novel mutations.

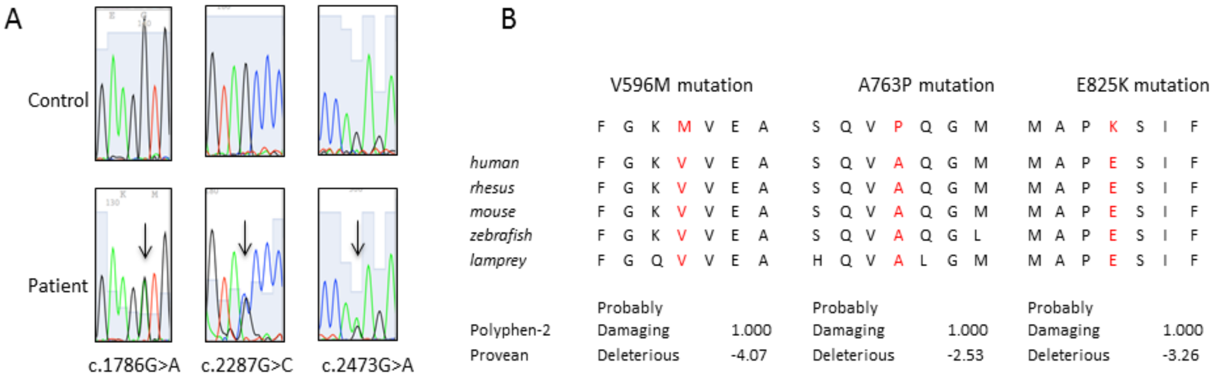

Supplement: Web supplement [file jnnp-2015-310788-s1.pdf]
