# Supplementary material for: Hereditary leukoencephalopathy with axonal spheroids: a spectrum of phenotypes from CNS vasculitis to parkinsonism in an adult onset leukodystrophy series
Source: J Neurol Neurosurg Psychiatry. 2015 May 2;87(5):512–9. doi: 10.1136/jnnp-2015-310788 (PMC4853550; doi:10.1136/jnnp-2015-310788)
Supplement: Web table 1 [file jnnp-2015-310788-s2.pdf]

Supplementary Table 1: Summary of all reported CSF1R mutations, exons, age at onset and age at death.

Author	Year	Mutation	Exon	Age at onset	Age at death	Disease duration
Rademakers[1]	2011	G589E	13	47	58	11
Rademakers	2011	G589E	13	58	61	3
Rademakers	2011	E633K	14	42	46	4
Rademakers	2011	E633K	14	67	74	7
Rademakers	2011	E633K	14	78	84	6
Rademakers	2011	M766T	17	18	N/A	N/A
Rademakers	2011	M766T	17	43	N/A	N/A
Rademakers	2011	I755N	17	48	N/A	N/A
Rademakers	2011	A770P	17	52	63	11
Rademakers	2011	I794T	18	35	N/A	N/A
Rademakers	2011	F849del	19	63	67	4
Rademakers	2011	D837Y	19	N/A	N/A	N/A
Rademakers	2011	F849S	19	N/A	N/A	N/A
Rademakers	2011	P878T	20	39	49	10
Rademakers	2011	P878T	20	39	43	4
Rademakers	2011	M875T	20	41	N/A	N/A
Rademakers	2011	M875T	20	46	N/A	N/A
Rademakers	2011	L868P	20	55	63	8
Rademakers	2011	M875T	20	58	N/A	N/A
Rademakers	2011	M875T	20	71	N/A	N/A
Rademakers	2011	M875T	20	N/A	N/A	N/A
Rademakers	2011	Gly585_lys619delinsAla	IVS12	36	40	4
Rademakers	2011	Gly585_lys619delinsAla	IVS12	38	41	3
Rademakers	2011	C774_N814delins	IVS18	23	N/A	N/A
Rademakers	2011	cys774_asn814del	IVS18	50	55	5
Kinoshita[2]	2012	R782H	18	51	N/A	N/A
Kleinfeld[3]	2013	I794T	18	37	N/A	N/A
Kleinfeld	2013	F828S	19	39	N/A	N/A
Kleinfeld	2013	F828S	19	47	N/A	N/A
Kondo[4]	2013	K793T	18	40	N/A	N/A
Mitsui[5]	2012	C653Y	14	43	N/A	N/A
Mitsui	2012	C653Y	14	48	N/A	N/A
Mitsui	2012	R777W	18	38	N/A	N/A
Mitsui	2012	I794T	18	52	N/A	N/A
Mitsui	2012	I794T	18	60	N/A	N/A
Mitsui	2012	R777W	18	65	N/A	N/A
Ahmed[6]	2013	A781V	18	32	N/A	N/A
Ahmed	2013	A781V	18	54	N/A	N/A

Ahmed	2013	A781V	18	58	N/A	N/A
Ahmed	2013	A781V	18	60	N/A	N/A
Ahmed	2013	A781V	18	67	N/A	N/A
Fujioka[7]	2013	G589E	13	46	N/A	N/A
Fujioka	2013	G589E	13	47	N/A	N/A
Fujioka	2013	G589E	13	58	N/A	N/A
Fujioka	2013	G589E	13	59	N/A	N/A
Guerreiro[8]	2013	T567fsX44	12	24	N/A	N/A
Guerreiro	2013	L630R	14	45	N/A	N/A
Guerreiro	2013	E633K	14	46	N/A	N/A
Guerreiro	2013	R777Q	18	44	N/A	N/A
Guerreiro	2013	R777W	18	46	N/A	N/A
Guerreiro	2013	I794T	18	46	N/A	N/A
Guerreiro	2013	R777Q	18	58	N/A	N/A
Guerreiro	2013	R777Q	18	60	N/A	N/A
Guerreiro	2013	L817P	19	36	N/A	N/A
Guerreiro	2013	I827T	19	44	N/A	N/A
Guerreiro	2013	E847D	19	44	N/A	N/A
Guerreiro	2013	Y856H	20	39	N/A	N/A
Guerreiro	2013	Y856H	20	42	N/A	N/A
Guerreiro	2013	Y856H	20	42	N/A	N/A
Guerreiro	2013	Y856H	20	47	N/A	N/A
Guerreiro	2013	Y856H	20	55	N/A	N/A
Guerreiro	2013	P901S	21	20	N/A	N/A
Guerreiro	2013	c2655-2A>G	IVS20	50	N/A	N/A
Inui[9]	2013	A777Gly	18	24	N/A	N/A
Karle[10]	2013	I794T	18	29	N/A	N/A
Karle	2013	R777Q	18	38	N/A	N/A
Karle	2013	I794T	18	40	N/A	N/A
Karle	2013	A781V	18	50	N/A	N/A
Karle	2013	I843N	19	40	N/A	N/A
Karle	2013	V838L	19	53	N/A	N/A
Karle	2013	Q877X	20	28	N/A	N/A
Nicholson[11]	2013	M766T	17	51	N/A	N/A
Nicholson	2013	R782H	18	N/A	N/A	N/A
Saitoh[12]	2013	I794T	18	28	N/A	N/A
Sundal[13]	2013	G589E	13	58	N/A	N/A
Sundal	2013	E633K	14	42	N/A	N/A
Sundal	2013	E633K	14	67	N/A	N/A
Sundal	2013	M766T	17	18	N/A	N/A
Sundal	2013	M766T	17	27	N/A	N/A

Sundal	2013	M766T	17	43	N/A	N/A
Sundal	2013	I755Asn	17	48	N/A	N/A
Sundal	2013	A770P	17	52	N/A	N/A
Sundal	2013	I794T	18	35	N/A	N/A
Sundal	2013	I794T	18	57	N/A	N/A
Sundal	2013	M875T	20	58	N/A	N/A
Sundal	2013	M875T	20	71	N/A	N/A
Sundal	2013	Gly585_lys619delinsAla	IVS12	36	N/A	N/A
Sundal	2013	Gly585_lys619delinsAla	IVS12	38	N/A	N/A
Sundal	2013	cys774_asn814del	IVS18	23	N/A	N/A
Sundal	2013	cys774_asn814del	IVS18	50	N/A	N/A
Terasawa[14]	2013	A823V	19	52	N/A	N/A
Battisti[15]	2014	C653R	14	48	N/A	N/A
Battisti	2014	I843F	19	56	N/A	N/A
Battisti	2014	I1906T	21	37	N/A	N/A
Hoffmann[16]	2014	R777Q	18	22	N/A	N/A
Hoffmann	2014	R777Q	18	29	N/A	N/A
Hoffmann	2014	R777Q	18	30	N/A	N/A
Hoffmann	2014	R777Q	18	40	N/A	N/A
Kitani Morii[17]	2014	I794T	18	N/A	N/A	N/A
Konno[18]	2014	S688Efx13	15	41	N/A	N/A
Konno	2014	G765D	17	37	N/A	N/A
Konno	2014	A781E	18	36	N/A	N/A
Konno	2014	I794T	18	40	N/A	N/A
Konno	2014	I794T	18	55	N/A	N/A
Konno	2014	P842S	19	45	N/A	N/A
Konno	2014	C2442+1G>T	IVS18	53	57	4
La Piana[19]	2014	V784M	18	39	N/A	N/A
Levin[20]	2014	V838L	19	N/A	N/A	N/A
Schuberth[21]	2014	L582P	12	54	N/A	N/A
Schuberth	2014	E633K	14	41	N/A	N/A
Schuberth	2014	V838L	19	45	N/A	N/A
Sundal[22]	2015	Asn854Lys	20	29	N/A	N/A
Sundal	2015	Asn854Lys	20	38	N/A	N/A
Sundal	2015	Asn854Lys	20	42	N/A	N/A
Sundal	2015	Asn854Lys	20	44	N/A	N/A
Sundal	2015	Asn854Lys	20	45	N/A	N/A
Sundal	2015	Asn854Lys	20	50	N/A	N/A
Kim [23]	2015	M766V	17	39	N/A	N/A
Kim	2015	R782H	18	37	42	5
Kim	2015	A781V	18	41	N/A	N/A

Kim	2015	A781V	18	44	49	5
Lynch	2015	V596M	13	31	36	5
Lynch	2015	E633K	14	29	N/A	N/A
Lynch	2015	A763P	17	45	51	6
Lynch	2015	G825L	19	42	N/A	N/A
Lynch	2015	c2442+1 G>a	IVS18	44	N/A	N/A

SUPPLEMENTARY REFERENCES

1. Rademakers, R. *et al.* Mutations in the colony stimulating factor 1 receptor (CSF1R) gene cause hereditary diffuse leukoencephalopathy with spheroids. *Nat. Genet.* **44,** 200–205 (2011).

2. Kinoshita, M., Yoshida, K., Oyanagi, K., Hashimoto, T. & Ikeda, S. I. Hereditary diffuse leukoencephalopathy with axonal spheroids caused by R782H mutation in CSF1R: Case report. *J. Neurol. Sci.* **318,** 115–118 (2012).

3. Kleinfeld, K. *et al.* Adult-onset leukoencephalopathy with neuroaxonal spheroids and pigmented glia: report of five cases and a new mutation. *J. Neurol.* **260,** 558–71 (2013).

4. Kondo, Y., Kinoshita, M., Fukushima, K., Yoshida, K. & Ikeda, S. Early involvement of the corpus callosum in a patient with hereditary diffuse leukoencephalopathy with spheroids carrying the de novo K793T mutation of CSF1R. *Intern. Med.* **52,** 503–6 (2013).

5. Mitsui, J. *et al.* CSF1R mutations identified in three families with autosomal dominantly inherited leukoencephalopathy. *Am. J. Med. Genet. B. Neuropsychiatr. Genet.* **159B,** 951–7 (2012).

6. Ahmed, R. *et al.* A novel A781V mutation in the CSF1R gene causes hereditary diffuse leucoencephalopathy with axonal spheroids. *J. Neurol. Sci.* **332,** 141–144 (2013).

7. Fujioka, S. *et al.* An adult-onset leukoencephalopathy with axonal spheroids and pigmented glia accompanied by brain calcifications: a case report and a literature review of brain calcifications disorders. *J. Neurol.* **260,** 2665–8 (2013).

8. Guerreiro, R. *et al.* Genetic analysis of inherited leukodystrophies: genotype-phenotype correlations in the CSF1R gene. *JAMA Neurol.* **70,** 875–82 (2013).

9. Inui, T. *et al.* A new CSF1R mutation presenting with an extensive white matter lesion mimicking primary progressive multiple sclerosis. *J. Neurol. Sci.* **334,** 192–5 (2013).

10. Karle, K. N. *et al.* De novo mutations in hereditary diffuse leukoencephalopathy with axonal spheroids (HDLS). *Neurology* **81,** 2039–2044 (2013).

11. Nicholson, A. M. *et al.* CSF1R mutations link POLD and HDLS as a single disease entity. *Neurology* **80,** 1033–1040 (2013).

12. Saitoh, B.-Y. *et al.* A case of hereditary diffuse leukoencephalopathy with axonal spheroids caused by a de novo mutation in CSF1R masquerading as primary progressive multiple sclerosis. *Mult. Scler.* **19,** 1367–70 (2013).

13. Sundal, C. *et al.* Parkinsonian features in hereditary diffuse leukoencephalopathy with spheroids (HDLS) and CSF1R mutations. *Park. Relat. Disord.* **19,** 869–877 (2013).

14. Terasawa, Y. *et al.* Increasing and persistent DWI changes in a patient with hereditary diffuse leukoencephalopathy with spheroids. *J. Neurol. Sci.* **335,** 213–5 (2013).

15. Battisti, C. *et al.* Hereditary diffuse leukoencephalopathy with axonal spheroids: three patients with stroke-like presentation carrying new mutations in the CSF1R gene. *J. Neurol.* **261,** 768–72 (2014).

16. Hoffmann, S. *et al.* Enlarging the nosological spectrum of hereditary diffuse leukoencephalopathy with axonal spheroids (HDLS). *Brain Pathol.* (2014). doi:10.1111/bpa.12120

17. Kitani-Morii, F. *et al.* Hereditary Diffuse Leukoencephalopathy with Spheroids Characterized by Spastic Hemiplegia Preceding Mental Impairment. *Intern. Med.* **53,** 1377–1380 (2014).

18. Konno, T. *et al.* Haploinsufficiency of CSF-1R and clinicopathologic characterization in patients with HDLS. *Neurology* **82,** 139–48 (2014).

19. La Piana, R., Webber, A., Guiot, M.-C., Del Pilar Cortes, M. & Brais, B. A novel mutation in the CSF1R gene causes a variable leukoencephalopathy with spheroids. *Neurogenetics* **15,** 289–94 (2014).

20. Levin, J. *et al.* Diffuse leukoencephalopathy with spheroids: biopsy findings and a novel mutation. *Clin. Neurol. Neurosurg.* **122,** 113–5 (2014).

21. Schuberth, M. *et al.* [Hereditary diffuse leukencephalopathy with spheroids: a microgliopathy due to CSF1 receptor impairment]. *Nervenarzt* **85,** 465–70 (2014).

22. Sundal, C. *et al.* Hereditary diffuse leukoencephalopathy with spheroids with phenotype of primary progressive multiple sclerosis. *Eur. J. Neurol.* **22,** 328–33 (2015).

23. Kim, E.-J. *et al.* Adult-onset leukoencephalopathy with axonal spheroids and pigmented glia linked CSF1R mutation: Report of four Korean cases. *J. Neurol. Sci.* **349,** 232–8 (2015).
