# Supplementary material for: Hereditary leukoencephalopathy with axonal spheroids: a spectrum of phenotypes from CNS vasculitis to parkinsonism in an adult onset leukodystrophy series
Source: J Neurol Neurosurg Psychiatry. 2015 May 2;87(5):512–9. doi: 10.1136/jnnp-2015-310788 (PMC4853550; doi:10.1136/jnnp-2015-310788)
Supplement: Web table 2 [file jnnp-2015-310788-s3.pdf]

Supplementary Table 2: Summary of the clinical presentations of the 43 patients in whom a *CSF1R* mutation was not found.

| Case | Sex | Age at onset (y) | Clinical Features | | | | | MRI Features | Additional |
|---|---|---|---|---|---|---|---|---|---|
| | | | Cognitive Decline | Behavioural Change | Seizures | Spasticity | Ataxia | WML | |
| **1** | m | 28 | - | - | - | + | - | + | |
| **2** | f | 25 | - | - | + | - | - | + | |
| **3** | f | 43 | + | + | - | + | - | + | |
| **4** | f | 36 | - | - | - | + | - | + | Vertigo, migraine |
| **4** | f | 53 | + | + | - | - | - | + | |
| **5** | m | 33 | + | + | - | - | - | + | AD Family History |
| **6** | m | 51 | + | - | - | - | - | + | AD Family History |
| **7** | f | 42 | + | - | - | - | - | + | |
| **8** | f | 55 | + | + | - | - | - | + | |
| **9** | m | 57 | + | + | - | - | - | + | AD Family History |
| **10** | m | 18 | + | + | - | + | - | + | |
| **11** | m | 52 | + | + | - | + | - | + | Myoclonus |
| **12** | m | 35 | - | - | - | - | - | + | Migraine |
| **13** | f | 76 | - | - | - | + | - | + | Lower limb sensory disturbance |
| **14** | m | 57 | + | + | - | - | + | + | |
| **15** | f | 21 | - | - | - | + | + | + | |
| **16** | m | 18 | - | + | - | - | - | + | Upper limb tremor |
| **17** | m | 46 | + | - | - | - | - | + | |
| **18** | m | 64 | + | - | - | - | + | + | Myoclonus |
| **19** | m | 30 | - | - | - | + | + | + | |

| | | | | | | | | | |
|---|---|---|---|---|---|---|---|---|---|
| **20** | m | 64 | - | - | - | + | + | + | |
| **21** | f | 53 | + | - | - | + | - | + | Vertigo |
| **22** | m | 18 | + | + | - | - | - | + | |
| **23** | m | 50 | + | + | - | - | - | + | |
| **24** | f | 47 | - | - | - | - | + | + | |
| **25** | f | 42 | + | + | - | - | - | + | AD Family History |
| **26** | m | 60 | + | - | - | - | + | + | |
| **27** | f | 57 | + | + | - | - | + | + | |
| **28** | m | 37 | + | + | - | - | - | + | |
| **29** | f | 18 | - | - | + | + | - | + | |
| **30** | m | 44 | - | - | - | - | - | + | Visual Change, dysarthria |
| **31** | f | 44 | + | - | - | - | + | + | Dysarthria |
| **32** | m | 45 | - | - | - | + | + | + | |
| **33** | m | 18 | + | + | - | + | - | + | Myoclonus |
| **34** | m | 22 | + | + | - | - | - | + | |
| **35** | f | 22 | + | - | - | - | - | + | |
| **36** | f | 44 | + | - | + | + | - | + | |
| **37** | f | 56 | + | - | - | - | - | + | |
| **38** | m | 23 | + | - | - | - | - | + | |
| **39** | m | 34 | + | - | - | - | - | + | |
| **40** | f | 68 | + | - | - | - | - | + | |
| **41** | f | 35 | + | - | - | + | - | + | |
| **42** | m | 42 | - | - | - | - | - | + | |
| **43** | f | 54 | - | - | + | + | - | + | |

WML: Symmetric T2 hyperintense white matter lesions, m: male, f: female, +: present, -:absent, AD: Autosomal Dominant
